# Hypoionic shock treatment enables aminoglycosides antibiotics to eradicate bacterial persisters

**DOI:** 10.1038/srep14247

**Published:** 2015-10-05

**Authors:** Liu Jiafeng, Xinmiao Fu, Zengyi Chang

**Affiliations:** 1 State Key Laboratory of Protein and Plant Gene Research, School of Life Sciences, Peking University, Beijing 100871, China; 2 Center for Protein Science, Peking University, Beijing 100871, China

## Abstract

Bacterial persisters, usually being considered as dormant cells that are tolerant to antibiotics, are an important source for recurrent infection and emergence of antibiotic resistant pathogens. Clinical eradication of pathogenic persisters is highly desired but greatly difficult mainly due to the substantial reduction in antibiotics uptake as well as the non-active state of the drug targets. Here we report that bacterial persisters (normal growing cells as well) can be effectively eradicated by aminoglycoside antibiotics upon hypoionic shock (e.g. pure water treatment) even for less than one minute. Such hypoionic shock potentiation effect on aminoglycosides is proton motive force-independent, and is apparently achieved by promoting the entrance of aminoglycosides, speculatively through the mechanosensitive ion channels. Our revelations may provide a simple and powerful strategy to eradicate pathogen persisters.

Bacterial pathogens insusceptible to antibiotics, either due to “resistance” or “tolerance”, are a growing threat to public health worldwide^1^,^2^,^3^,^4^. While resistance is generally caused by acquisition of resistance genes or mutation of the target genes^5^,^6^, tolerance is usually due to the bacteria cells entering a non-growing dormant state, commonly defined as persisters, rather than any genetic alteration^7^,^8^,^9^. As a result, the antibiotics targets are largely in a non-active state and meanwhile the uptake of certain antibiotics (such as aminoglycosides) hardly occurs^10^,^11^, both of which make antibiotics inefficient for eradicating pathogen persisters. Additionally, such bacterial persisters may provide a reservoir of pathogens that facilitate the subsequent evolution of antibiotic resistance^12^,^13^,^14^. Because of these, eradication of bacterial persisters, though being highly desired, is greatly challenging in technical terms.

Aminoglycosides, as represented by streptomycin, tobramycin, kanamycin, gentamicin and amikacin, are commonly used antibiotics against many threatening infectious diseases (e.g. tuberculosis) and are known to act by binding and inhibiting bacterial ribosome^15^,^16^. A metabolism recovery-based approach was recently explored in killing bacterial persisters by enhancing proton-motive force-dependent uptake of aminoglycosides^11^. Here, we attempted to develop a method to efficiently eradicate bacterial persisters also by aminoglycosides, but independent of metabolism recovery.

## Results

### Hypoionic shock potentiate aminoglycoside antibiotics to eradicate *E. coli* persisters

**Figure 1:**
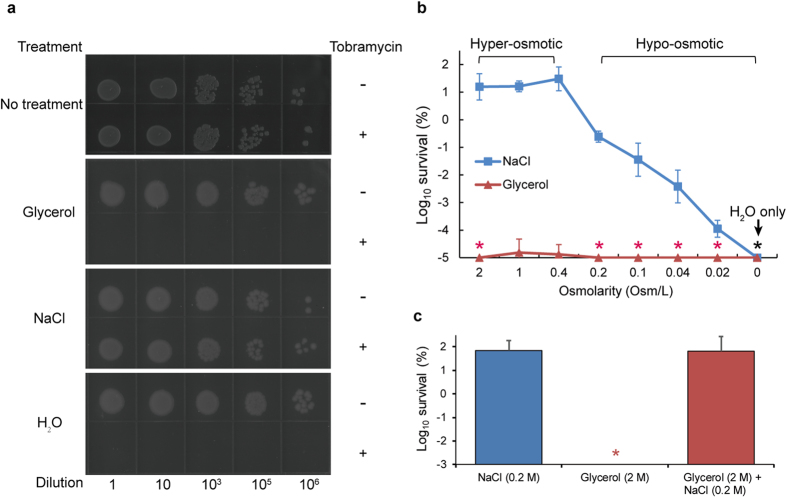
Hypoionic shock potentiate aminoglycoside antibiotics to eradicate *E. coli* persisters. (**a**) Plated stationary phase *E. coli* cells after being treated for 1 hour with tobramycin-containing (500 μg/ml) solutions of glycerol (2 M), NaCl (1 M) or distilled water. Data are representative of three independent experiments. (**b,c**) Survival ratio (in log scale) of the stationary phase *E. coli* cells after being treated for 1 hour with tobramycin-containing (500 μg/ml) solutions of the indicated osmolarity of NaCl or glycerol (**b**) or solution of mixed NaCl and glycerol (**c**). The data in (**b**,**c**) represent mean ± s.d. of three independent experiments. Asterisks indicate no detection of survival. The osmolarity of the LB medium is estimated to be around 0.4 Osm/L.

During our exploration, we tested whether such physicochemical stress conditions as osmotic shock, among others, would help to promote the effect of aminoglycosides for killing persisters. In this regard, the stationary phase *E. coli* cells, which are highly insusceptible to such antibiotics as aminoglycosides (Fig. 1a), were also used as model persisters as before^11^,^17^,^18^. We strikingly found that the bacterial persisters were almost completely eradicated after being treated for one hour with tobramycin-containing solution of 2 M glycerol, but not of 1 M NaCl, although both of which theoretically have an identical osmolarity (i.e., osmotic concentration) (Fig. 1a). This apparently indicates that the bactericidal effect of aminoglycosides observed with the 2 M glycerol treatment could not be simply due to a hyper-osmotic shock effect. Consistent with this analysis, we observed similar bactericidal effects of tobramycin dissolved in solutions of a wide spectrum of glycerol concentrations, from hyper-osmotic to iso-osmotic to hypo-osmotic (Fig. 1b). It was reported that glycerol, at a concentration of 20 mM and dissolved in M9 salts, is able to potentiate aminoglycosides against bacterial persisters^11^. Here, glycerol is unlikely to act in a similar way, because of its far higher concentration and also the lack of other essential salts for growth. In addition, we also demonstrated that tobramycin-containing solutions of non-metabolizable osmolyte DMSO^19^ or xylitol^20^ exhibited similar eradication effect (Supplementary Fig. 1). Remarkably, for the effect of NaCl, we noticed that the bactericidal efficiency of tobramycin increases upon a decrease of NaCl concentration, achieving an eradiation effect when NaCl became completely absent, i.e., only in the presence of water (Figures 1a, 1b).

These observations indicate that the effect of our treatment to potentiate tobramycin to eradicate persisters is apparently attributed to the lowering of ionic strength of the treating solutions. In this regard, we observed that adding NaCl at a concentration as low as 0.2 M could abolish the potentiation effect of the glycerol solution on tobramycin (Fig. 1c). In general, we found that treating in non-electrolyte (as glycerol) solutions exhibits similar potentiation effect for tobramycin to eradicate persisters, while treating in strong electrolyte (as NaCl) solutions hardly exhibits any such potentiation effect (Supplementary Fig. 1). Our dosage effect analyses reveal that upon such hypoionic shock treatment, about 99.9% of the stationary phase cells were killed in the presence of tobramycin at a concentration of 25 μg/ml, and achieving an effective eradication at 500 μg/ml (Supplementary Fig. 2). Furthermore, such hypoionic shock are apparently effective for aminoglycoside antibiotics in general, as similarly observed for streptomycin and kanamycin, but not for the other two major types of bactericidal antibiotics, either β-lactams (as represented by ampicillin) that inhibit cell wall synthesis, or quinolones (as represented by ofloxacin) that inhibit DNA synthesis (Supplementary Fig. 3). Taken together, these observations implicate that hypoionic (i.e. low ionic strength) shock treatment specifically promote the aminoglycoside type of antibiotics to eradicate *E. coli* persisters.

### The potentiation effect of hypoionic shock on aminoglycoside antibiotics is instant and independent of proton-motive force

**Figure 2:**
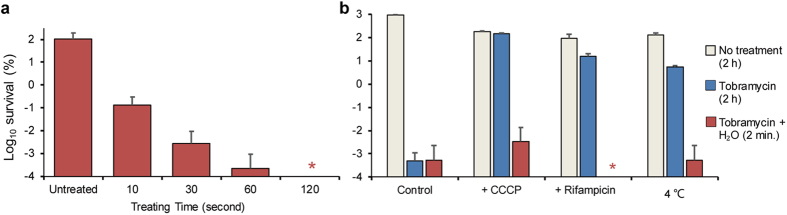
The potentiation effect of hypoionic shock on aminoglycoside antibiotics is instant and independent of proton-motive force. (**a**) Survival ratio of stationary phase *E. coli* cells after being treated with tobramycin-containing (500 μg/ml) distilled water for the indicated time. (**b**) Exponential phase *E. coli* cells pre-treated for 1 hour with CCCP (20 μM) or rifampicin (100 μg/ml), or placed at 4 °C became tolerant to the antibiotics tobramycin. The survival ratio of such cells after being treated with tobramycin-containing (25 μg/ml) distilled water for 2 minutes was then measured. The data represent mean ± s.d. of three independent experiments. Asterisks indicate no detection of survival.

We next demonstrated that, to be effective, tobramycin has to be present during the hypoionic shock treatment, while adding it afterwards becomes hardly effective (Supplementary Fig. 4). This suggests that the potentiation effect is generated during but not after the hypoionic shock treatment. Our further analysis indicate that *E. coli* persisters could be almost completely killed after being treated for as short as 2 minutes (or about 99.9% killed even after only 10 seconds treatment) by tobramycin under our hypoionic shock condition (Fig. 2a). It is noteworthy that similar short-time exposure of tobramycin under hypoionic shock treatment was found to be able to eradicate exponential phase non-persister *E. coli* cells, which otherwise may take hours of tobramycin exposure to achieve similar eradication effect^15^ (Figure 2b, Supplementary Fig. 5). Consistently, a two-minute hypoionic shock treatment was also found to enable tobramycin to efficiently eradicate conditioned *E. coli* persister cells (Fig. 2b), which were made insusceptible to antibiotics by treating exponential phase cells with bacteriostatic compound rifampicin (being an transcription inhibitor) or placing the cells at a low temperature of 4 °C (both for one hour)^7^. Furthermore, we demonstrated that the potentiation effect of short-time hypoionic shock treatment for aminoglycoside antibiotic to eradicate *E. coli* persisters was found to be independent of the proton-motive force across the plasma membrane (Supplementary Fig. 6), as the persister-killing effect largely remained even in the presence of carbonyl cyanide m-chlorophenyl hydrazone (CCCP), an uncoupler that was previously reported to eliminate the proton-motive force and to abolish the uptake of aminoglycosides^11^,^15^. Taken together, these observations apparently indicate that the potentiation effect of hypoionic shock on aminoglycoside antibiotics against persister cells is instant and independent of the proton-motive force.

### Ribosomes are possibly the target for aminoglycoside antibiotics to eradicate persisters upon hypoionic shock treatment

**Figure 3:**
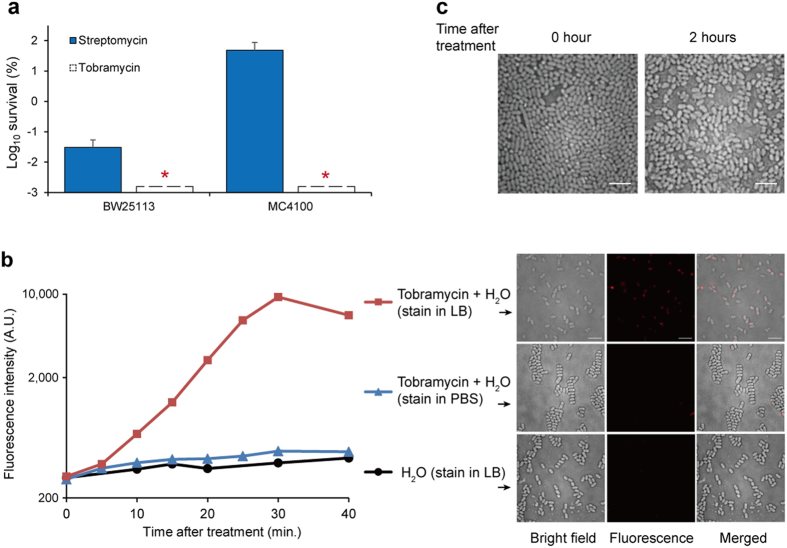
The bacterial cell death likely occurs upon the reawakening of the persisters. (**a**) Survival ratio of stationary phase MC4100 (streptomycin-resistant) or BW25113 (streptomycin-sensitive) *E. coli* cells after being treated with tobramycin- (500 μg/ml) or streptomycin-containing (2 mg/ml) distilled water. Asterisks indicate no detection of survival. (**b**) Time-dependent fluorescence-activated cell sorting analysis (left) and direct fluorescent microscopy imaging (right; at the 30 minutes point) of stationary phase cells that were treated with tobramycin-containing (500 μg/ml) distilled water before incubated with propidium iodide (50 μg/ml) in PBS or LB for the indicated length of time. A.U., arbitrary units. (**c**) Regular micrographs of the cells taken at the indicated time point. Scale bar, 5 μm.

We next found that, upon such hypoionic shock treatment, the aminoglycoside antibiotics kill persister *E. coli* cells still by inhibiting the 30S ribosome subunit, being their conventional target^15^. In particular, we demonstrated that the persister cells of the *E. coli* strain MC4100, which is resistant only to streptomycin but not to other aminoglycoside antibiotics (e.g. tobramycin) due to the occurrence of a unique point mutation on the 30S ribosomal subunit protein S12^21^, could no longer be eradicated by streptomycin even with our hypoionic shock treatment (Fig. 3a). This observation suggests that the aminoglycoside antibiotics apparently have entered the cells upon the hypoionic shock. In support of our conclusion, it was reported about half a century ago that the entrance of streptomycin-^14^C into exponentially growing *E. coli* cells was significantly enhanced after being diluted with distilled water^22^. Further, it was reported before that the uptake of aminoglycosides into bacterial cells is irreversible^23^. It follows that the cell death most likely occurs due to and upon the reawakening of the persisters, when the killing effect of the antibiotics is realized.

In support of this conclusion, we observed that the persister cells treated with tobramycin under hypoionic shock condition started to die soon after being incubated in antibiotics-free LB growth medium but not in phosphate buffer saline (PBS), as demonstrated either by time-dependent fluorescence-activated cell sorting analysis or direct fluorescent microscopy imaging (at 30 minutes staining) after the dead cells being stained with the fluorescent dye propidium iodide (Fig. 3b). The misshaping of the cells that were plated in LB agarose growth medium for 2 hours was also clearly observed by regular microscopy (Fig. 3c).

### The aminoglycoside antibiotics is hypothesized to enter the persister cells through the mechanosensitive ion channels upon the hypoionic shock treatment

In retrospect, the hypo-osmotic shock (a type of hypoionic shock) was reported to activate bacterial mechanosensitive ion channels, being non-specific and passive ion transporters located on the plasma membrane^24^,^25^,^26^. It was recently reported that the potency of aminoglycoside streptomycin depends on the expression of a mechanosensitive ion channel in *E coli* cells^27^. In this regard, our observation that the potentiation effect could be strongly suppressed by such ions as NaCl (Fig. 1c; Supplementary Fig. 1) suggests one possibility that the aminoglycosides enters the bacteria cells through mechanosensitive ion channels upon our hypoionic shock treatment. It has been reported that such mechanosensitive ion channels open only for a short moment upon hypo-osmotic shock treatment^28^, which apparently explains our observation that adding the aminoglycosides after the shock becomes hardly effective (Supplementary Fig. 4). It should be pointed out that the diameter of an active mechanosensitive channel could be as large as 30 Å^24^,^29^, which would allow such small molecules as aminoglycosides (with a molecule mass of about 500 Dalton) to effectively pass through.

Nevertheless, in a preliminary study, we failed to observe any significant decrease of the hypoionic shock-promoted bactericidal effect of tobramycin against persister cells when each of the seven *E. coli* genes encoding the mechanosensitive channels was individually deleted (Supplementary Fig. 7), most likely due to the functional redundancy of the mechanosensitive channels^30^. We did not perform this study using mutants having a combined deletion of two or more genes encoding the mechanosensitive channels, because such combined mutation was reported to decrease the viability of the bacteria against hypo-osmotic shock^26^,^31^. Evidently, such mutants are inappropriate for testing our hypoionic shock-promoted bactericidal effect of tobramycin.

**Figure 4:**
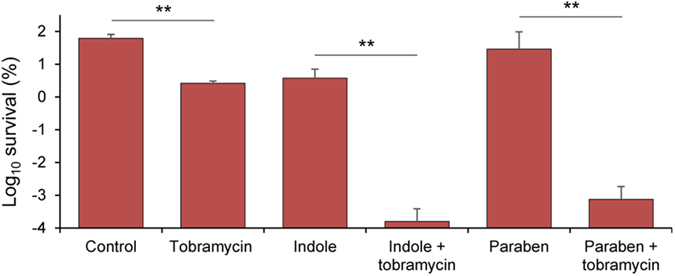
Aminoglycoside antibiotics enter persister cells likely through mechanosensitive ion channels under hypoionic shock condition. Survival ratio of stationary phase Gram-positive *S. aureus* bacterial cells after being treated with tobramycin-containing (500 μg/ml) distilled water in the presence of 1 mM indole or 1 mM propyl-paraben. Cells treated with indole- or paraben-containing distilled water serves as the control. The data represent mean ± s.d. of three independent experiments. **P < 0.01.

Furthermore, we found that hypoionic shock treatment also significantly promoted the bactericidal effect of tobramycin against conditioned persister cells of Gram-positive bacterium *S. aureus*, which were prepared by treating the exponential phase cells with bacteriostatic antibiotics rifampicin or placing the cells at a low temperature of 4 °C (Supplementary Fig. 8). We found that tobramycin exhibited only mild eradication effect to the *S. aureus* stationary phase cells upon such hypoionic shock treatment (Fig. 4). This inefficiency might reflect the fact that they possess thicker cell wall in comparison with either the exponential phase cells^32^ or the stationary phase cells of Gram-negative bacteria^33^, such that their mechanosensitive ion channels may become less sensitive to hypoionic shock. To the *S. aureus* cells, we found that the presence of indole or propyl-paraben, each being a chemical activator of mechanosensitive ion channels^34^,^35^, greatly increased the bactericidal effect of tobramycin under hypoionic shock conditions (Fig. 4), which is consistent with our hypothesis that the aminoglycoside antibiotics may enter the persister cells through the mechanosensitive ion channels. Nevertheless, our data here do not rule out the possibility that the potentiation effect is generated through a nonspecific increase of the permeability of the cell membrane.

## Discussion

**Figure 5:**
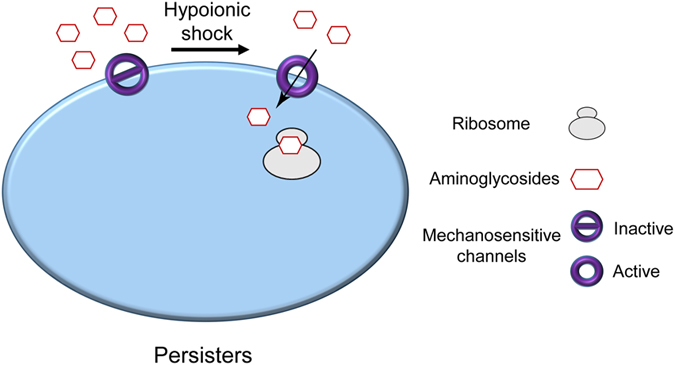
Hypothetic illustration for hypoionic shock-promoted entrance of aminoglycoside antibiotics into bacteria cells. Based on our hypothesis, the mechanosensitive ion channels are directly activated by membrane tension upon hypoionic shock treatment, and the ionic aminoglycoside antibiotics instantly enter the persister bacteria cells down the concentration gradient. Such entrance of aminoglycoside antibiotics is irreversible and lead to bacteria cell death due to and upon cell re-awakening.

In summary, here we report a simple and effective method for eradicating bacterial persisters (normal growing cells as well) such that the instant entrance of aminoglycoside antibiotics is promoted by a hypoionic shock treatment. Our findings implicate a possibility that enhancing the activity of pathogens’ mechanosensitive ion channels by physical (e.g. hypoionic shock) and/or chemical (e.g. indole and parabens) manipulations may in general increase the bactericidal efficiency of the aminoglycoside type of antibiotics (as illustrated in Fig. 5). In clinical terms, our strategy may help to fight against the infectious pathogens with increasing tolerance and resistance towards antibiotics, particularly for infections in exposed areas of the body (e.g. the skin and the surface of alimentary canal). One challenging issue for the potential application of our method is how to avoid the suppressing effect of ions naturally present in the infected sites.

## Methods

### Antibiotics and chemicals

For treating exponential phase bacterial cells, the concentrations of antibiotics were used as follows: Ampicillin 50 μg/ml, kanamycin 50 μg/ml, tobramycin 25 μg/ml, ofloxacin 5 μg/ml, streptomycin 100 μg/ml, rifampicin 100 μg/ml. For treating stationary phase cells, all concentrations were 20 folds higher in view of the much higher cell density. Carbonyl cyanide m-chlorophenyl hydrazone (CCCP) (20 μM), propidium iodide (PI) (50 μg/ml), indole (1 mM), propyl 4-hydroxybenzoate (paraben) (1 mM) and all the antibiotics were of analytical grade.

### Bacterial strains, media and growth conditions

Stationary phase Gram-negative *Escherichia coli* bacteria cells of strains BW25113 and MC4100 or Gram-positive *Staphylococcus aureus* bacteria cells of the strain ATCC 25923 were prepared by overnight culturing frozen stock bacteria cells at 37 °C, 200 r.p.m. in 20 ml of Luria-Bertani (LB) growth medium. For preparing exponential phase cells, the stationary phase cells were diluted 1:500 in 20 ml LB growth medium and further cultured at 37 °C, 200 r.p.m. to an OD600 of ~0.4–0.6.

### Cell treatment

A volume of 100 μl exponential phase (about 2 × 10^7^ cells) or stationary phase (about 5 × 10^8^ cells) cultured bacteria were harvested by centrifugation at 10,000 g for 1 minute, resuspended in 100 μl of selected tobramycin-containing solutions, incubated for a specified time, washed and serially diluted in PBS, before spotted on LB plates for survival ratio measurements. The colony forming units (CFU) were counted after the cells were incubated overnight at 37 °C.

### Conditioned persister cells

They are prepared by treating exponential phase bacterial cells with 100 μg/ml rifampicin or being placed at 4 °C for 1 hour.

### Dead cell staining

Stationary phase *E. coli* cells (100 μl) were harvested and treated with 100 μl tobramycin-containing (500 μg/ml) distilled water, before washed and incubated at room temperature (20 °C) in PBS or LB, each containing propidium iodide (PI) (50 μg/ml). At a given time point, samples were analyzed on a BD Accuri C6 flow cytometer with the following settings: detector, FL2; flow rate, 14 μl/min.; core size, 10 μm.

### Microscopy

Samples were plotted on agarose after PI staining. Images were recorded at 30 °C with an N-SIM imaging system (Nikon) equipped with a 100X/1.49 NA oil-immersion objective (Nikon) and laser beams (561 nm).

### Statistical methods

Each survival ratio analysis was performed with samples collected from three independent cultures and data were presented as mean ± standard deviation. The survival ratio was calculated by dividing the CFU number of the experimental (treated) group by that of the control (untreated) group. The two-tailed Student’s *t*-test was used to calculate the *p*-values for certain experiments (Fig. 4). For fluorescence-activated cell sorting (FACS) analysis (Fig. 3b), the data represent mean fluorescence intensity of ~50,000 cells (the same pattern was observed in three independent experiments).

## Additional Information

**How to cite this article**: Jiafeng, L. *et al.* Hypoionic shock treatment enables aminoglycosides antibiotics to eradicate bacterial persisters. *Sci. Rep.* **5**, 14247; doi: 10.1038/srep14247 (2015).

## Supplementary Material

Supplementary Information
