# Supplementary material for: Hypoionic shock treatment enables aminoglycosides antibiotics to eradicate bacterial persisters
Source: Sci Rep. 2015 Oct 5;5:14247. doi: 10.1038/srep14247 (PMC4593029; doi:10.1038/srep14247)
Supplement: Supplementary Information [file srep14247-s1.pdf]

Supplementary information

Hypoionic shock treatment enables aminoglycosides antibiotics to eradicate bacterial persisters

Liu Jiafeng[1], Xinmiao Fu[1,2,*], and Zengyi Chang[1,2,*]

[1]State Key Laboratory of Protein and Plant Gene Research, School of Life Sciences, Peking University, Beijing 100871, China

[2]Center for Protein Science, Peking University, Beijing 100871, China

[*]Corresponding. Changzy@pku.edu.cn or fuxinmiao@pku.edu.cn

Supplementary Figures

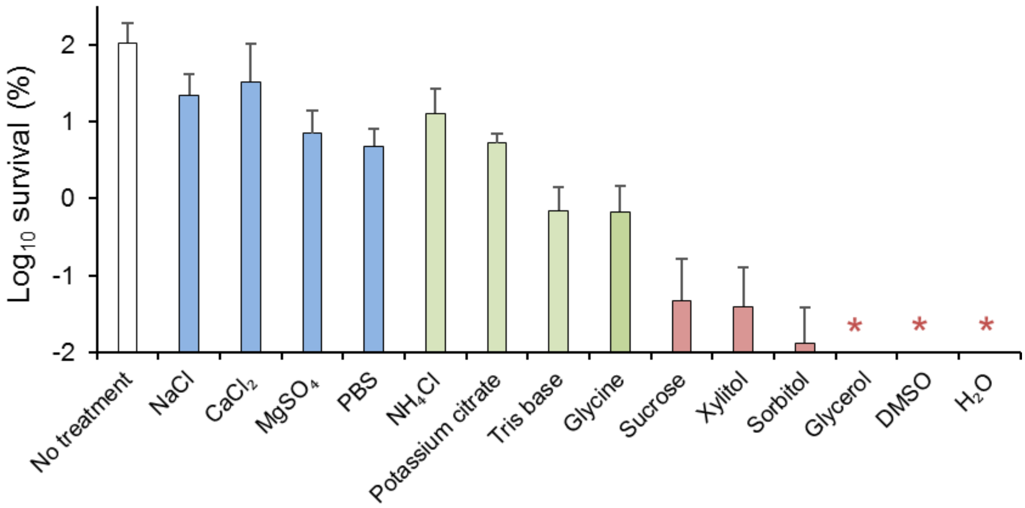

Supplementary Figure 1. Persister cells can be eradicated by aminoglycoside antibiotics only upon hypoionic shock treatment. Survival ratio of stationary phase *E. coli* cells after being treated with distilled water containing tobramycin (500 µg/ml) and the indicated solutes all at an osmotic concentration of about 0.4 Osm/L, i.e., being roughly the same as that of the original LB medium.

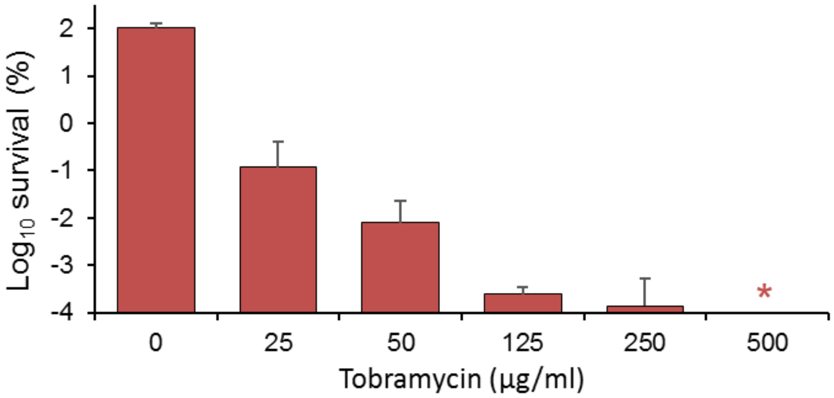

Supplementary Figure 2. Dosage effect of aminoglycosides to eradicate persister cells under hypoionic shock condition. Survival ratio of stationary phase *E. coli* cells were measured after being treated for 2 minutes with distilled water containing the indicated concentrations of tobramycin.

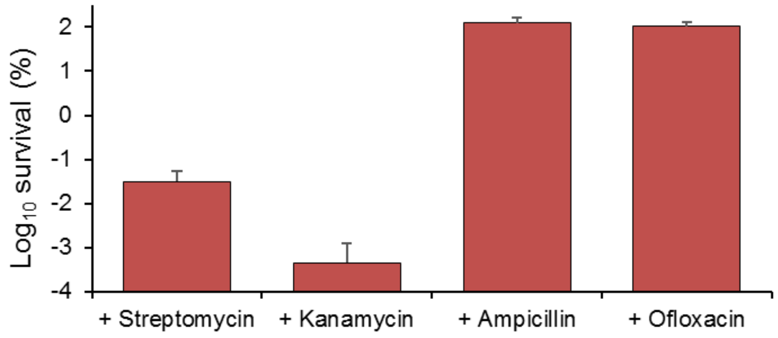

Supplementary Figure 3. Hypoionic shock-potentiated eradication of persisters is specific only to aminoglycoside antibiotics. Survival ratio of stationary phase E. coli cells were measured after being treated with distilled water containing streptomycin (2 mg/ml), kanamycin (1 mg/ml), ampicillin (1 mg/ml) or ofloxacin (100 μg/ml), all being 20 folds of the concentration that effectively kill the exponential phase cells.

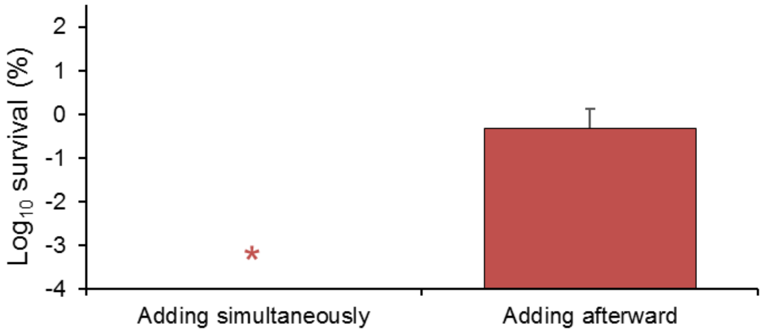

Supplementary Figure 4. Adding tobramycin after the hypoionic shock treatment would greatly reduce its bactericidal effect against persister cells. Survival ratio of stationary phase *E. coli* cells after being treated simultaneously with distilled water and tobramycin (500 μg/ml), or being treated with distilled water for two minutes before tobramycin was added.

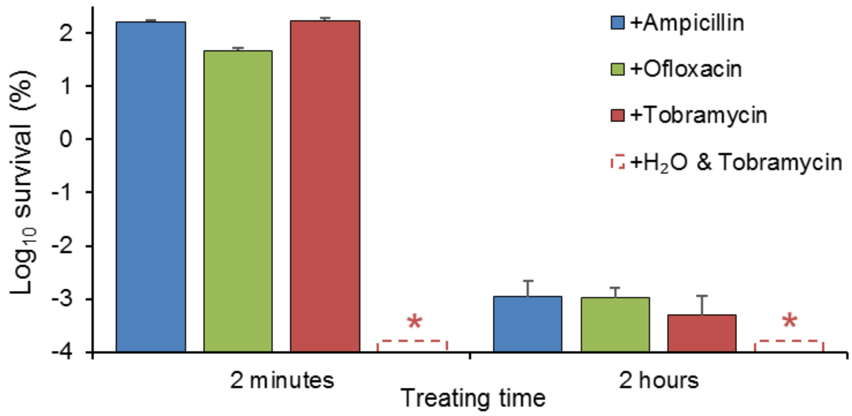

Supplementary Figure 5. Hypoionic shock potentiate aminoglycoside antibiotics to instantly eradicate *E.coli* non-persisters. Survival ratio of exponential phase *E. coli* cells after being treated with the indicated antibiotics for the indicated time.

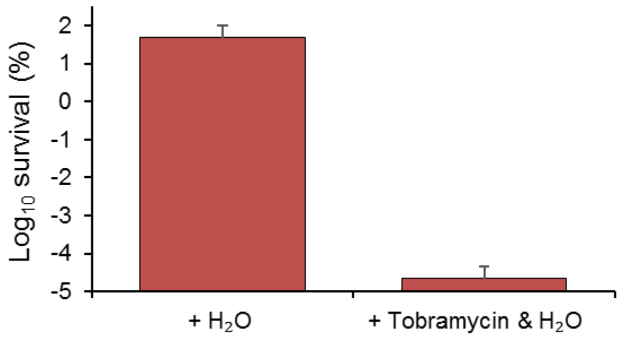

Supplementary Figure 6. Proton gradient uncoupler CCCP does not significantly decrease the killing efficiency of aminoglycoside antibiotics against persister cells under hypoionic shock condition. Stationary phase *E. coli* cell cultures were supplemented with CCCP (at a final concentration of 20 μM) and further incubated for 1 hour, followed by treating for 2 minutes with tobramycin-containing (500 μg/ml) distilled water before cell survival ratio was measured.

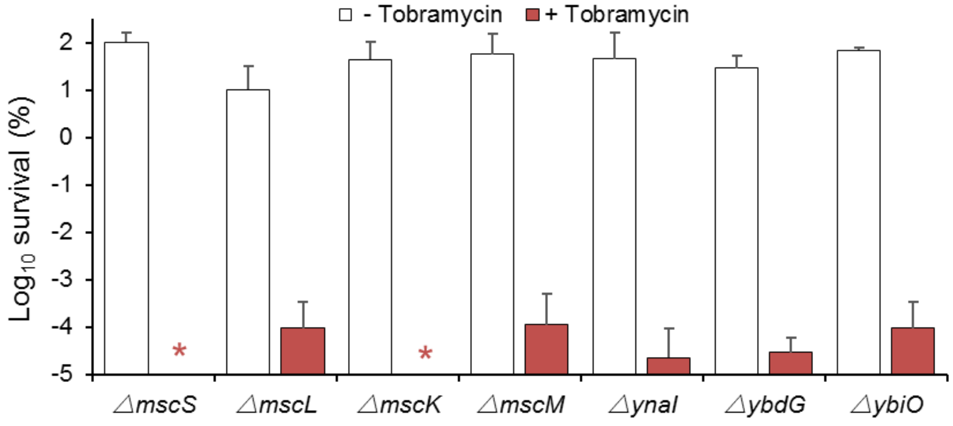

Supplementary Figure 7. No significant decrease of the hypoionic shock enhanced bactericidal efficiency of tobramycin against persister cells when each of the seven genes encoding the mechanosensitive channels was individually deleted. Survival ratio of the stationary phase cells of single mechanosensitive channel knockout strain of *E. coli* after being treated with distilled water containing 500 μg/ml tobramycin.

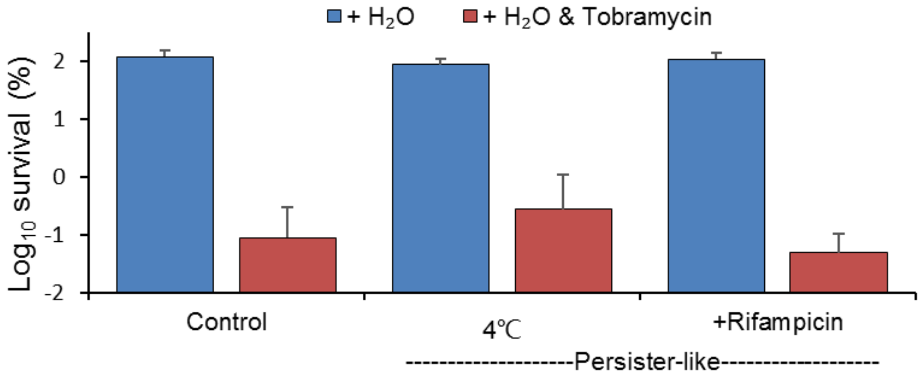

Supplementary Figure 8. Hypoionic shock potentiates aminoglycoside antibiotics to kill both growing and non-growing persister-like *S. aureus* cells. The conditioned persister *S. aureus* (non-growing) cells that were prepared by incubating the exponential phase cells with rifampicin (100 µg/ml) or placing at 4℃ (both for 1 hour), were treated with distilled water containing 25 µg/ml tobramycin before the survival ratio was measured.
